# Supplementary material for: Effects of Pretreatment and Polarization Shielding on EK-PRB of Fe/Mn/C-LDH for Remediation of Arsenic Contaminated Soils
Source: Nanomaterials (Basel). 2023 Jan 12;13(2):325. doi: 10.3390/nano13020325 (PMC9860780; doi:10.3390/nano13020325)
Supplement: Supplementary file 1 [file nanomaterials-13-00325-s001.zip › nanomaterials-2128926-supplementary.pdf]

nanomaterials

MDPI

Supplementary Materials

Effects of Pretreatment and Polarization Shielding on EK-PRB of Fe/Mn/C-LDH for Remediation of Arsenic Contaminated Soils

Zongqiang Zhu [1,2,3], Yusong Kong [1], Hongqu Yang [4], Yan Tian [1], Xiaobin Zhou [1,2,*], Yinian Zhu [1,2], Zhanqiang Fang [5], Lihao Zhang [1], Shen Tang [1] and Yinming Fan [1,2,3,*]

[1] Collaborative Innovation Center for Water Pollution Control and Water Safety in Karst Area, Guilin University of Technology, Guilin 541004, China
[2] The Guangxi Key Laboratory of Theory and Technology for Environmental Pollution Control, Guilin University of Technology, Guilin 541004, China
[3] Technical Innovation Center of Mine Geological Environmental Restoration Engineering in Southern Karst Area, Nanning 530022, China
[4] Chongqing Hechuan Ecology and Environment Monitoring Station, Chongqing 401519, China
[5] School of Chemistry and Environment, South China Normal University, Guangzhou 510006, China
* Correspondence: zhouxiaobin@glut.edu.cn (X.Z.); yming_fan2015@163.com (Y.F.)

S1. Supplementary Texts

S1.1. Materials

S1.1.1. Soil

The sampling depth of soil sampling was 5–20 cm, and dried naturally after removing stones and plant residues. The dried soil was sieved through 20 meshes and fully mixed with a certain concentration of arsenic solution. The target arsenic contaminated soil was obtained after aging and culturing for one month with a moisture content of about 35%.

S1.1.2. Preparation of PRB Filler Fe/Mn/C-LDH

In this study, the waste bamboo scraps from the bamboo processing industry in Guangxi were selected as the original structural template. Through a series of modification processes such as pretreatment, precursor solution leaching, washing, drying and muffle furnace calcination, the PRB material for the remediation of arsenic-contaminated soil by electrically driven enhanced permeable reactive wall was prepared. The specific preparation method is as follows:

Fe/Mn/C-LDH composites were prepared by co-precipitation method at a constant pH value using 100 mesh sieved bamboo charcoal as a template.

1. Preparation of bamboo charcoal: (i) The bamboo was cut into blocks of about 30 mm × 10 mm × 3 mm, and immersed in 5.00 % dilute ammonia water for 6 h at 100 °C. The bamboo after soaking was washed with deionized water and dried in an oven at 80 °C for 24 h. (ii) The dried bamboo was placed in a muffle furnace and calcined at 600 °C for 3 h. Sample 1 was obtained by grinding and passing through a 100-mesh sieve. (iii) The obtained sample 1 was immersed in concentrated nitric acid and boiled in an oil bath at 110 °C for 2 h, then washed with deionized water until the pH value was neutral, and dried at 80 °C to obtain a bamboo charcoal template.
2. Preparation of Fe/Mn/C-LDH by co-precipitation method: (i) According to the molar ratio of Fe:Mn of 2:1, quantitative ferric chloride and manganese chloride were weighed and dissolved in ultrapure water to obtain a mixed solution of iron and manganese with a concentration of 1 mol/L. (ii) The prepared bamboo charcoal template was immersed in ultrapure water to make it fully wet. The pH value of the solution system was adjusted to 11.5 with 3.2 M sodium hydroxide and 1M sodium carbonate solution. (iii) Fe-Mn mixed solution and mixed alkali solution were added dropwise to the mixed solution of bamboo charcoal template with a pipette to obtain

the mixed solution system for preparing materials. During the preparation process, the pH value of the mixed solution system was controlled at 11.5. (iv) The mixed solution was stirred for 2 h and then placed in a water bath at 60 °C for 24 h. (v) The solution was taken out and cooled to room temperature, and it was filtered and washed until the pH was neutral. Then it was freeze-dried. The dried product was Fe/Mn/C-LDH material.

S1.2 Construction and Operation of EK-PRB Device

The experimental reactor of this study is a self-made EK-PRB device. The schematic diagram is shown in Figure 1 and the physical diagram is shown in Figure S1. The whole EK-PRB device is customized from organic glass. The main components of the device include a DC power supply, soil filling chamber, electrolysis chamber, electrolyte collection tank, circulating pump and electrode plate.

1. Soil chamber: length × width × height (10.0 cm × 5.0 cm × 5.0 cm). Both ends of the soil chamber are connected to the cathode and anode electrolysis chamber, and the middle is separated by a perforated organic glass plate. When filling the soil, a 300 mesh filter cloth should be placed between the soil and the partition, and a PRB material wrapped by the filter cloth should be placed in the middle of the soil. The type and filling amount of the material can be determined according to the type of pollutant and the amount of remediation.
2. Electrolysis chamber: length × width × height (5.0 cm × 5.0 cm × 5.0 cm), separated from the soil chamber by a perforated organic glass plate. There is a clamping slot in the middle, and different electrode plates can be placed. The upper and lower ends of the outer side of the electrolytic chamber are respectively provided with a pipe orifice with a diameter of 1.0 cm, which can not only ensure the water level balance of the electrolytic chamber and the soil chamber but also maintain the concentration of the electrolyte unchanged by externally connecting the electrolyte collection tank.
3. Electrolyte collection tank: 500 mL capacity, collect the electrolyte overflowed from the electrolysis chamber, and connects the reflux peristaltic pump through the peristaltic tube to make the electrolyte of the collection tank continuously flow in the electrolysis chamber. Control the water level and solution concentration of the electrolysis chamber.

S1.3Analysis Methods

S1.3.1. Soil Physical and Chemical Properties

The pH value and electrical conductivity (EC) of the treated soil were measured by pH and EC meters (pHS-3E, Shanghai, China) after extraction with deionized water using a soil/water ratio of 1:2.5 (pH) and 1:5 (EC). The redox environment of arsenic in soil was described by measuring the redox potential of soil pore waters [1].

S1.3.2. Analysis Method for Arsenic Content

In detail, 2.5 g of dry soil was mixed with 50 mL TCLP extraction, and oscillated at 25 ℃ for 18 ± 2 h. Besides, to determine the total and residual arsenic content, 0.2 g of dry soil was digested in 10 mL of aqua regia in a boiling water bath for 2 h. The arsenic concentration and leaching toxicity concentration were analyzed by inductively coupled plasma spectrometer (Optima 7000DV, Waltham, MA, USA) [2].

S2. Supplementary Tables

Table S1. The physical and chemical properties of soil before and after pollution.

Soil	pH	EC (μs/cm)	Eh (mV)	Organic Matter (g/kg)	Alkali Hydrolyzed Nitrogen (mg/kg)	Available Phosphorus (mg/kg)	Target Arsenic Concentration (mg/kg)
Background soil	5.35	50.12	422	25.31	91.35	9.01	-
Arsenic contaminated soil	6.35	268	381	-	-	-	500

* The selection of target heavy metal pollution concentration was based on the field investigation data of Guangxi contaminated site remediation project; * "—" represented not detected.

Table S2. The extraction of heavy metals from soil by improved BCR method.

Arsenic Form	Extraction	Experimental Condition	Soil/Water
Acid dissolved state	20 mL 0.1 M HOAc	Oscillated at 25 ℃ for 16 h	1:40
Reducible state	20 mL 0.5 M $NH_4OH \cdot HCl$	Oscillated at 25 ℃ for 16 h	1:40
Oxidizable state	10 mL 8.8 M H_2O_2<br>50 mL1 M NH_4OAc	Water bath heating at 85 ℃	1:10
Residue state	HCl~HNO_3~HF~$HClO_4$	Electric heating plate digestion	——

S3. Supplementary Figures

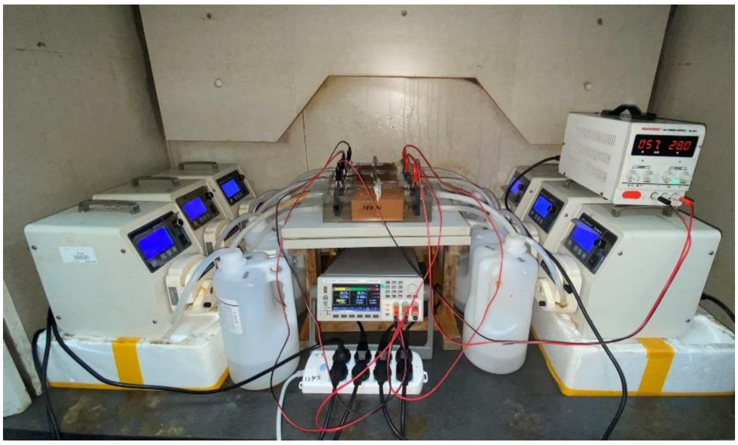

Figure S1. Electric drive enhanced permeable reactive wall device diagram.

References

1. Xu, Y.; Li, J.; Xia, W.; Sun, Y.; Qian, G.; Zhang, J. Enhanced remediation of arsenic and chromium co-contaminated soil by eletrokinetic-permeable reactive barriers with different reagents. *Environ. Sci. Pollut. Res.* **2018**, *26*, 3392–3403, https://doi.org/10.1007/s11356-018-3842-9.
2. Yuan, C.; Chiang, T.-S. Enhancement of electrokinetic remediation of arsenic spiked soil by chemical reagents. *J. Hazard. Mater.* **2008**, *152*, 309–315, https://doi.org/10.1016/j.jhazmat.2007.06.099.
